# Perinatal Exposure to Phthalates: From Endocrine to Neurodevelopment Effects

**DOI:** 10.3390/ijms22084063

**Published:** 2021-04-14

**Authors:** Laura Lucaccioni, Viola Trevisani, Erica Passini, Beatrice Righi, Carlotta Plessi, Barbara Predieri, Lorenzo Iughetti

**Affiliations:** 1 Pediatric Unit, Department of Medical and Surgical Sciences of the Mothers, Children and Adults, University of Modena and Reggio Emilia, 41124 Modena, Italy; barbara.predieri@unimore.it (B.P.); lorenzo.iughetti@unimore.it (L.I.); 2 Department of Medical and Surgical Sciences of the Mothers, Post Graduate School of Paediatrics, Children and Adults, University of Modena and Reggio Emilia, 41124 Modena, Italy; viola.trevisani@gmail.com (V.T.); erica.passini11@gmail.com (E.P.); 3 Pediatric Unit, Ramazzini Hospital, 41012 Carpi, Italy; beatrice.righi2406@gmail.com; 4 Department of Medical and Surgical Sciences and NeuroSciences, Post Graduate School of Pediatric Surgery, University of Siena, 53100 Siena, Italy; carlotta.plessi@hotmail.it

**Keywords:** phthalates, endocrine disrupting chemicals (EDCs), prenatal exposure, endocrine endpoints, neurodevelopment, newborn

## Abstract

Phthalates, as other endocrine disrupting chemicals (EDCs), may alter the homeostasis and the action of hormones and signaling molecules, causing adverse health outcomes. This is true especially for infants, who are both more exposed and sensitive to their effects. Phthalates are particularly harmful when the exposure occurs during certain critical temporal windows of the development, such as the prenatal and the early postnatal phases. Phthalates may also interfere with the neuroendocrine systems (e.g., thyroid hormone signaling or metabolism), causing disruption of neuronal differentiation and maturation, increasing the risk of behavioral and cognitive disorders (ADHD and autistic behaviors, reduced mental, psychomotor, and IQ development, and emotional problems). Despite more studies being needed to better understand the role of these substances, plenty of evidence suggests the impact of phthalates on the neuroendocrine system development and function. This review aims to update the knowledge on the neuroendocrine consequences of neonatal and perinatal exposure to phthalates.

**Background**: Phthalates are a group of plasticizers classified as endocrine-disrupting chemicals (EDCs) by the National Institute of Health, the World Health Organization, and other agencies. Since these compounds are widely spread, humans may encounter EDCs on a daily basis and during all stages of life, from conception and fetal development to adulthood and senescence [1]. However, prenatal and early postnatal lapses are the most critical temporal windows of the child development. During fetal life, the placental barrier is not completely impermeable to the passage of harmful substances and the environmental exposure could affect and permanently reprogram normal physiological responses [2]. In fact, the “Developmental Origin of Health Disease” (DOHaD) hypothesis postulates that early life adverse environments can cause epigenetic shifts, promoting the development of different diseases in adulthood [1,3]. The aim of this narrative review is to point out the more recent knowledge covering the effect of early exposure to phthalates on pediatric wellbeing, with a specific focus on the consequences of the prenatal and neonatal exposure on endocrine and neurological human systems. **Methods:** A literature search was conducted using PubMed CENTRAL databases. All searches used the terms “phthalates” OR “endocrine disrupting chemicals” AND “prenatal exposure” OR “neonate” OR “neuroendocrine development” OR “endocrine endpoints”. The publications included in the review were basic research studies, randomized trials, cohort studies, and reviews from 2000 to 2020, with a specific focus on publications between 2015 and 2020. The research was limited to papers published in English. The reference lists of retrieved studies have also been reviewed to identify studies that may have not been spotted by the search strategy. We excluded all the studies with insufficient statistical analysis, possible biases, and not clear end-points. Our main focus was for in vivo human studies, although many animal in vivo and in vitro studies were also included to better explain some of the mechanisms of action of phthalates in humans. 

## 1. Phthalates Classification and Metabolism Pathway

Phthalates are esters of 1,2-benzenedicarboxylic acid and their chemical structure consists of one benzene ring linked with two ester functional groups. According to their molecular weight, phthalate esters (PEs) are classified into two distinct groups: high-molecular-weight phthalates (HMW) and low-molecular-weight phthalates (LMW). 

HMWs (diisodecyl phthalate (DiDP), diisononyl phthalate (DiNP), di2-propylheptyl phthalate (DHPH), diisoundecyl phthalate (DiUP), and diisotridecyl phthalate (DTDP)), are widely used in industry as plasticizers to improve softness, elongation, and durability of rigid polymers and in particular polyvinyl chloride (PVC). They can be found in wires and cables, flooring, truck tarpaulins, wall coverings, self-adhesive films or labels, synthetic leather, coated fabrics, roofing membranes, and automotive applications [4]. Low-molecular-weight (LMW) phthalates (dibutyl phthalate (DBP), diisobutyl phthalate (DiBP), butyl benzyl phthalate (BBP), and di 2-ethylhexyl phthalate (DEHP)) can be used in PVC products as well as in medical devices, adhesives, paints, inks, and enteric-coated tablets. 

Dimetyl phthalate (DMP) and diethyl phthalate (DEP) are not classified within these groups because they are not plasticizers and not related to PVC. They are mainly used in personal care products such as cosmetics, fragrances, and medical devices [5].

Since phthalates do not establish a permanent bond with the plastic products, under temperature variation and pH influence they can migrate to the environment [6]. Due to their ubiquitous presence in the environment, humans can be exposed to phthalates through various pathways (ingestion, inhalation, injection, or absorption through the skin) [7,8,9]. When phthalates enter the human body, they are distributed by the circulatory system and quickly metabolized by intestinal lipase and esterases [10]. The metabolic reactions involve hydrolysis of the diesters to their respective monoesters, oxidation, and conjugation with glucuronic acid. DMP, DEP, and BBzP are excreted without oxidation as their hydrolytic metabolites (MMP, MEP, and MzBP, respectively). DBP and DiBP are excreted mainly as the hydrolytic monoester, while DEHP and DiNP are eliminated mainly as oxidative metabolites [11,12,13].

The half-life for some of these compounds is estimated to be less than 10 h (MEP, < 6 h; MEHHP, 10 h) [14,15]. The pathways of excretion are via biological fluids (saliva and semen), urine, sweat, meconium, and breastmilk [10,11,12,16]. 

As for every chemical product, daily exposure to phthalates could be related to risks for health effects. The tolerable daily intake (TDI) dose is an estimate of the amount of a single chemical in food or drinking water that can be assumed every day over a lifetime without a significant health risk. TDI is expressed as milligrams per kilo of body weight (bw) per day [17]. The current estimated levels of human exposure to phthalates have been declared safe by US and EU food safety agencies [18,19].

For phthalates, the TDI values established by EFSA for some of the most common diesters, such as DEHP, BBP, and DBP, are 0.05, 0.5 and 0.01 mg/kg bw/day, respectively, while for HMW molecules such as DiDP and DiNP, the TDI is 0.15 mg/Kg bw/day. Exceeding those amounts may increase the risk for reproductive effects for the LMW molecules group and liver effects for the HMWs [20].

Despite the ubiquitous exposure to phthalates through ingestion, inhalation, and skin absorption, TDIs may vary within the same population being influenced by consumer habits. Moreover, occupational-level exposure to phthalates may also influence the daily intake of specific categories of workers, although this is still a matter of debate. In a recent systematic review, Fréry et al. underlined how recent data from human biomonitoring on old and new phthalates exposure in occupational workers are still missing [21]. More studies are needed to understand the entity of daily exposure to EDCs, taking into account the chance that absorption and effects may not follow a monotonic curve. Nonmonotonic effects increase the difficulties in establishing safe exposure ranges, especially during specific vulnerable temporal windows through life.

In fact, the exposure to EDC during the early stages of development impacts the susceptibility of individuals to various diseases later in life. Phthalates have been reported crossing the placental barrier and detected in amniotic fluid after maternal exposure, even in early pregnancy. At this age, the fetal liver’s detoxification system is still unable to convert these metabolites, which may be free to act as EDCs on fetal development [22]. 

## 2. The Epigenetic Effects of Phthalates

Given the simultaneous daily exposure to a cocktail of toxic substances, it is difficult to establish direct causality between exposure to EDCs and their effects on human health. To understand how phthalates may influence the future wellbeing of new generations, contributing to the development of “noncommunicable” diseases (NCDs), it is important to point out that the NCDs are multifactorial in origin, resulting from the interaction between genetic and environmental factors. Early life has a strong influence in human development, and in particular on lifelong patterns of health and disease. The prenatal (in utero) environment is now recognized as a key driver of NCDs risk later in life [23].

David Barker was one of the first scientist who laid the foundation of this idea: in the 80s and 90s, he clearly identified the period from conception to 2 years of life (the first 1000 days) as one of the key determinants of an individual’s lifelong health trajectory [24,25].

The theory was then expanded and deepened in the following years in many studies, and evidence accumulated over the decades supporting the hypothesis of the “The Development Origin of Health and Disease” (DOHaD). Through this paradigm, studies are increasingly identifying links between stressors during pregnancy and the risk to offspring of a myriad of NCDs, including cardiovascular diseases, metabolic disorders (e.g., obesity and diabetes mellitus), and neurocognitive problems [26]. Causal mechanisms between gestational exposures of the fetus and adverse health outcomes later in life are now emerging; in particular, it is known that exposure to environmental toxicants and stresses acts as “modifiers” on the genetic heritage (genome) through epigenetic modifications [26,27,28]. Such epigenetic modifications involve the heritable transmission of the regulatory components of gene expression patterns that are passed to the germline of the fetus in development. The three important mechanisms known to regulate gene transcription epigenetically are DNA methylation, histone modifications, and regulation by noncoding RNA [28,29]. The concept of DOHaD has been refined over the years and now it is also known as the “three-hit” model. In fact, each embryo starts his life with his own genetic heritage, and this is considered to be the first “hit.” Individual genetic heritage is influenced by the early life exposome (including several factors such as maternal stress, drugs, hypertension, and maternal exposure to persistent organic pollutants), and that is the second “hit.” Together, the genetic heritage and the early life exposome lead to a phenotype, which is susceptible to the third environmental “hit” later in life. While current preventive strategies for diseases such as cardio-metabolic pathologies focus on high-risk individuals in mid-life, DOHaD concepts offer a “primordial” preventive strategy to reduce disease in future generations by improving and protecting fetal and infant development [30,31].

**Figure 1:**
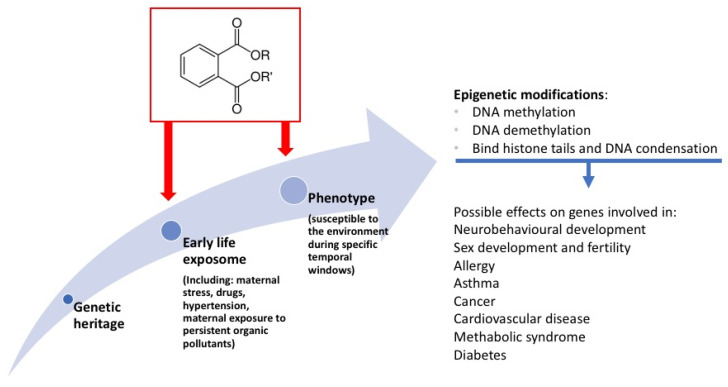
How phthalates may enter in The Development Origin of Health and Disease (DOHaD), causing epigenetic effects.

In fact, if the interference of epigenetic reprogramming occurs in germ cells, it will affect the epigenome and its progeny through a process called transgenerational epigenetic inheritance, even when descendants have no additional environmental exposure (second and third generations) [32]. Figure 1 summarizes the epigenetic role of phthalates.

It is important to bear in mind how children are more exposed to phthalates than adults are. Relatively higher levels of phthalate monoesters could be observed in young children compared with adults, who mainly excrete the long-branched phthalates as secondary metabolites, probably due to different metabolic pathways [33]. The immaturity of several liver detoxification systems during early neonatal and postnatal life emphasizes the importance of avoiding the early exposure to these chemicals, especially during pregnancy and childhood, to reduce the risks of future health consequences. 

Phthalates usually operate by modifying DNA methylation, demethylation, and chromatin expression, binding to histone tails and condensing DNA and noncoding RNA expression regulation [34,35,36]. Phthalates exposure throughout pregnancy may lead to a glucometabolic disfunction in adulthood and future generations, since the epigenetic pattern alters the function of critical genes. Animal models showed how the DEHP exposure in pregnant rats downregulates the expression of glucose transporter (Glut4), increasing the DNA methylation and tightening the chromatin structure of the promoter region of the gene [37]. Moreover, maternal exposure to DEHP in mice provoked a testicular dysfunction due to the DNA hypermethylation, inducing a downregulation of insulin-like hormone-3 (Insl3) [25]. Animal models showed how the methylation at the promoter regions of other imprinting genes, such as insulin-like growth factor 2 receptors (Igf2r) and paternally expressed gene 3 (Peg3) may influence pubertal abnormalities, ovarian disease, testis dysfunction, and obesity in adulthood, due to DEHP and DBP [36,37,38]. 

In humans, recent data highlight the significant effect the prenatal exposure to phthalates and their consequence on DNA methylation. Phthalate exposure in utero may affect the methylation pattern status of imprinted genes in newborn children, in particular for the following genes: a first group involved in androgen response *MEG3* [39], *PA2G4*, *HMGCR*, and *XRCC6*, all involved in androgen response [40], and a second group of genes involved in early birth delivery, such as *Alu* and *LINE1* [41]. The long-lasting effect of the epigenetic changes due to phthalates may lead to promote tumor progressions [42,43] and infertility [44].

## 3. Effects of Phthalate Exposure during Perinatal Life 

### 3.1. Exposure to Phthalates during the Prenatal Temporal Window and Effects on Pregnancy 

Limited but growing evidence suggests that the exposure to phthalates in utero could be associated to epigenetic modifications in genes involved in antiandrogenic effect, spermatogenesis, growth, diabetes, increased risk of cancer, as well as cell cycle, cell proliferation, protein secretion, and behavioral problems [3,45,46,47].

Moreover, in vitro studies showed how the exposure to bisphenol A and phthalates modifies the storage of lipids and the regulation of metabolism, activating peroxisome proliferator-activated receptors (PPAR) pathways both in mouse and in human cells, adipogenesis, and alteration of pancreatic β-cell function [48]. A prospective cohort study provided evidence that prenatal exposure to bisphenol A and certain variants of phthalates (DBP and DEHP metabolites) inversely associated with fetal adiponectin and leptin levels [49]; this could not only be related to birth outcome but could also modify body size and adiposity in early childhood [50].

PPARγ is also expressed in the human trophoblast and is essential for placenta development and function. Phthalate metabolites may regulate the trophoblastic differentiation acting at PPARγ. In fact, an in vivo animal study showed how a prenatal exposure to DEHP resulted in dose-dependent activation of PPARγ in rat placenta and led to changes in the expression of its downstream targets [51]; human studies are needed to better evaluate this effect.

Moreover, the in utero human exposure to phthalates increases the odds of delivering preterm neonates [45]. A recent study provided evidence that the exposure to DEP and phthalates’ polymers from drugs may cause an increased risk of preterm birth (PTB), underlining the importance of exposure to phthalates from pharmaceuticals, which could be easily avoided [52]. Another study analyzed the association of urinary concentrations of phthalate biomarkers and Bisphenol A (BPA) measured in early pregnancy with length of gestation in a cohort of women with no fertility problems, suggesting that early exposition to DEHP and other HMW phthalates could influence the duration of pregnancy, with consequences for neonatal and maternal health [53]. 

An increased attention on lifestyle improvement during pregnancy should be emphasized from doctors and midwifes, to better inform pregnant women about risks related to the introduction of phthalates in daily life. 

### 3.2. Exposure to Phthalates during the Prenatal Temporal Window: Anthropometric and Endocrine Outcomes of the Neonates

Adequate sex steroid hormone concentrations are essential for a normal fetal genital development in early pregnancy. Fetuses and newborns are more vulnerable to toxic chemicals because of their immature metabolism and their impaired ability to excrete the pollutants. Exposure to hormone mimickers during development can have permanent adverse health impacts that are noticeable at birth or later in life [54]. 

Some phthalates esters, such as DEHP, DBP, and BBP, have shown antiandrogenic effects or act as androgen receptor agonists. They may suppress or inhibit the biological effects of androgens and the normal body tissue response to these hormones. 

**Figure 2:**
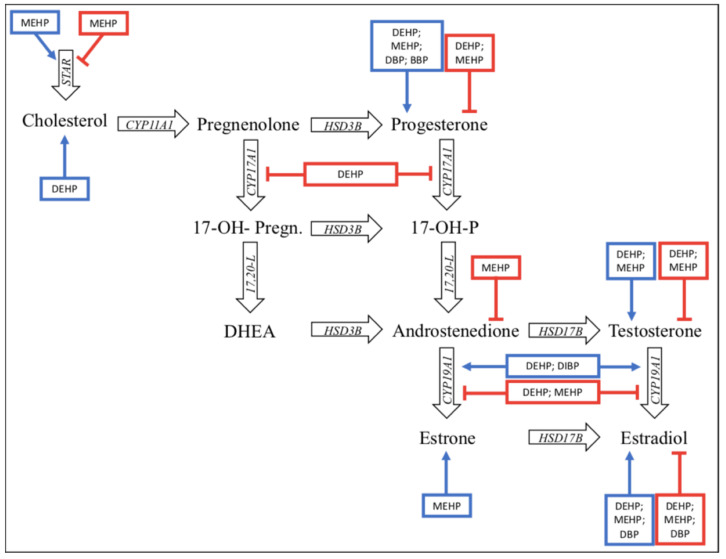
The major findings on the effects of phthalates on steroidogenesis are summarized. The phthalates metabolites modify the concentration of hormones and steroidogenic enzymes. Those in blue boxes increase the level of the hormone/steroidogenic enzyme indicated in black, while those in red boxes decrease the levels of the hormones/steroidogenic enzyme highlighted [55].

In particular, the downregulation of fetal testosterone production may alter the reproductive tract development. This issue was analyzed in animal models, evaluating the effect of phthalates on blood testosterone levels. The in utero exposure to anti-androgenic phthalates during the male programming window exerts a direct testicular toxic effect leading to a reduced testosterone production and, as consequence, to increased risk of developing conditions such as hypospadias and cryptorchidism. This is known as the “phthalate syndrome” (PS) in rats (Figure 2—[55]). The PS or at least one of the PS effects was found by Gray et al. in 2009 if the level of phthalate increased from 11 to 300 mg/kg/die in rats [56].

The measurement of endocrine-sensitive endpoints, especially those sexually dimorphic, such as the ano-genital distance (AGD), can be used as sentinels for later adverse health effects arising from prenatal exposure to endocrine-disrupting chemicals. The main known human effects are summarized in Table 1. Longer AGD in women has been associated with higher testosterone levels, possibly representing a masculinization effect in the prenatal period, while shorter AGD in males may imply diminished masculinization of the genitalia [57]. Some studies focused on the correlation between the duration of prenatal exposure to phthalates (DEHP) and the male infant penile size and AGD [58]. In particular, penile width, ano-scrotal distance, and ano-penile distance in male newborns were measured and modeled in relation to phthalate metabolite concentrations in maternal urine samples collected in each trimester of pregnancy (T1, T2, and T3). Penile width was inversely associated with T2-oxidized DEHP metabolites (MEOHP, MEHHP, and MECPP), although no appreciable associations were seen between penile width and T1 and T3 DEHP metabolite concentrations. Concentrations of DEHP metabolites in T1 urine samples were inversely related to male AGD [58,59]. Other studies evaluated the concentration of BPA and phthalate metabolites in the cord blood. BPA, DEHP, and MEHP levels were detectable in about 99% of cord blood samples. The levels of these substances were correlated to penile rod and AGD, and the data confirmed previous results: cord blood BPA is associated with a decrease in stretched penile length (SPL) in male newborns. In addition, they found that BPA increased cord blood estradiol levels while DEHP was significantly and inversely correlated with AGD level.

It has been proposed that male reproductive disorders, in particular those linked to the testicular dysgenesis syndrome (TDS), such as cryptorchidism, hypospadias, infertility, and testicular cancer, could be the result of ECDs exposure in utero [60]. Analyzing the association between genital anomalies and the concentration of urinary phthalates in the first trimester of pregnancy, the most frequent genital abnormality detected was hydrocele, followed by undescended testes and hypospadias. The phthalate metabolite majorly involved in these genital anomalies is DEHP. The maternal urinary levels of DEHP during the first trimester were associated with increased odds of developing hydrocele as well as any other genital anomaly in newborn males at birth. This may suggest a possible interference, with DEHP in causing a delayed closure of the processus vaginalis in the perinatal period [61].

Moreover, prenatal exposure to MEHP was associated with a reduction in the stretched length of the penis [45].

Phthalates could have sex-specific effects, since male and female newborns appear to be affected by different phthalates and in different ways [62].

**Table 1:** Human studies on endocrine effects of phthalates during prenatal life. DEHP: 2-Ethylhexyl phthalate; MEHP: mono(2-ethylhexyl) phthalate; AGD: anogenital distance; MEOHP: mono-(2-ethyl-5-oxohexyl) phthalate; MEHHP: mono (2-ethyl-5-hydroxyhexyl) phthalate; MECPP: methylerythritol cyclodiphosphate; LMW: low molecular weight phthalates.

Reference	Patients	Sex	Phthalates	Hypothetic Effect	Clinical Findings at Birth
Bustamante-Montes et al., 2013 [45]	73	Male	Maternal urinary level of MEHP collected during their last prenatal visit		reduction in the stretched length of the penis
Arbuckle T.E. et al., 2018 [57]	396 (198 girls)	Female	mono-benzyl phthalate (MBzP) and mono-ethyl phthalate (MEP) on urinary samples of the first trimester	Androgen and antiandrogen	Anoclitoris distance (ACD) was negatively associated with MBzP (feminizing) and positively associated with MEP (masculinizing).
	396 (198 boys)	Male	mono-n-butyl phthalate (MnBP) and the molar sum of low-molecular-weight phthalates on urinary samples of the first trimester	Androgen	Anopenile distance (APD) was positively associated with MnBP and ΣLMW
Sathyanarayana S. et al., 2016 [58]	371	Male	First trimester maternal urinary level of DEHP (75%ile = 3.63 μg/L)	delayed closure of the processus vaginalis	hydrocele, undescended testes and hypospadias
Sunman B. et al., 2019 [59]	100	Male	Cord blood DEHP		DEHP was inversely related to male AGD
Sathyanarayana S. et al., 2017 [61]	591	Male	oxidized DEHP metabolites (MEOHP, MEHHP, and MECPP) during the second trimester	Antiandrogen	Penile width was inversely associated with phthalates concentration
Barret ES et al., 2016 [63]	754 (370 boys)	Male	maternal urinary DEHP metabolite concentrations measured during the first trimester		inversely associated with AGD in male newborn
	754 (384 girls)	Female	maternal urinary DEHP metabolite concentrations measured during the first trimester		AGD measurments in girls were not associated with any maternal phthalate metabolite concentration

Furthermore, prenatal exposure to EDCs can alter the androgen activity with long-lasting health effects. In “The Infant Development and the Environment Study” (TIDES) [63], the largest study on phthalate exposure and AGD, maternal DEHP metabolite concentrations (molar sum: ∑DEHP = (MEHP*(1/278)) + (MEHHP*(1/294)) + (MEOHP*(1/292)) + (M ECPP*(1/308)) nmol/mL) measured during the first trimester (the critical period of time for androgen-related programming of the male reproductive system) were inversely associated with AGD in male newborns, while no association was found between prenatal phthalate exposure and female AGD.

Neonatologists, general pediatricians, and pediatric endocrinologists should start to focus on the higher incidence of mild forms of genital anomalies as a new clinical issue. A renewed clinical approach is needed, with early identification and careful clinical follow up for pediatric endocrinologists. 

### 3.3. Exposure to Phthalates during the Prenatal and Neonatal Temporal Window: Influence on Neurological Development

Several studies highlighted the potential role of EDCs as risk factors for neurodevelopmental impairment. The risk is higher when the exposure occurs during periods of increased vulnerability, such as prenatal, perinatal, and early postnatal life. This temporal window is known as the brain growth spurt (BGS) period. BGS starts during the third trimester of pregnancy and continues throughout the first two years of life. During the BGS period, the central nervous system is particularly sensitive to environmental stressors, since it is going through different critical developmental processes. Therefore, interfering with one or more of these processes (i.e., neuronal proliferation, migration, differentiation, synaptogenesis, and myelination) may result in a neurodevelopmental disruption affecting different behavioral domains (e.g., sensory, motor, and cognitive functions) [64,65].

Many are the mechanisms underlying the association between phthalates and developmental neurotoxicity. It is still not clear in which way pre- and neonatal exposure to phthalates affects their health later in life.

First of all, phthalate exposure may affect children’s health by causing oxidative stress. Ferguson et al. [66] reported statistically significant increases in oxidative stress biomarkers (i.e., 8-hydroxydeoxyguanosine (8-OHdG) and 8-isoprostane) in association with urinary phthalate metabolites (i.e., monobenzyl phthalate (MBzP), mono-n-butyl phthalate (MBP), and monoisobutyl phthalate (MiBP)) during pregnancy: it may be important for those pregnancy outcomes mediated by oxidative stress mechanisms and could be a contributory cause of neurodevelopmental problems. On one hand, 8-OHdG could cause oxidative DNA damage and apoptosis at the maternal–fetal interface [67], altering the placenta’s vascularization and consequently inducing preeclampsia and/or intrauterine growth restriction [68]. On the other hand, increased levels of prostaglandins, such as 8-isoprostane, may be involved in the preterm labor pathway, weakening the tissue’s integrity.

Moreover, phthalates might increase the risk of childhood neurodevelopmental disorders by interfering with different neuroendocrine systems. Table 2 summarizes the main human studies on prenatal phthalates exposure and possible neurobehavioral effects in the offspring.

Thyroid hormones play a critical role in neuronal migration, synaptogenesis, and myelination during fetal life and childhood [69]. A reduction of thyroxine during prenatal life is associated with deficits in visual processing, visual–motor abilities, and motor skills, while a postnatal reduction is associated with deficits in language, weaker fine motor skills, lower attention, and lack of memory [70,71]. Exposure to DBP seems to interfere with the thyroid function of pregnant women, since thyroxine (T4) and free T4 (FT4) were negatively associated with urinary levels of DBP. Altered maternal thyroid hormone levels during gestation have adverse effects on fetal neurodevelopment, which may be visible later in childhood [72].

The animal model presented by Xu et al. [73] investigated prenatal exposure to phthalates and modified expression of estrogen receptors in zebrafish. Their results showed that these compounds had a potential estrogenic activity and may inhibit neurogenesis in zebrafish during the embryonic stage. In fact, they revealed that both phthalates and a low concentration of estradiol inhibited the *esr2a* expression, causing a reduction of brain size as well as the number of mitotic neurons, corroborating the hypothesis that phthalates have a neurotoxicity action. Data on human brain size related to phthalates exposure are still missing. 

Prenatal exposure to DBP and DEHP has been related to poor attentional performances, aggressive behaviors, and oppositional and defiant problems. Moreover, experimental data showed that an early exposure to phthalates may disrupt structure and function of the hippocamus [74], a region of the brain associated with internalizing behaviors, such as anxiety and depression [75].

Recently, Daniel et al. [76] suggested that exposure to phthalates during prenatal life and early childhood affects children’s behavior in a sex-specific manner. In particular, they demonstrated that boys exposed to non-DEHP phthalates presented an anxious–shy behavior and that the concentration of DEHP metabolites was associated with decreased hyperactivity among girls.

**Table 2:** Phthalate neurological effects.

Reference	Patients	Sex	Phthalates	Effects	Outcome
Ferguson et al., 2019 [66]	130	Male and female	urinary phthalate metabolites: monobenzyl phthalate (MBzP), mono-n-butyl phthalate (MBP), and monoisobutyl phthalate (MiBP) during pregnancy	significant increases in oxidative stress biomarkers (i.e., 8-hydroxydeoxyguanosine (8-OHdG) and 8-isoprostane)	pregnancy outcomes mediated by oxidative stress mechanisms (DNA damage and apoptosis at the maternal–fetal interface, preeclampsia and/or intrauterine growth restriction, preterm labour pathway) and could be contributory cause of neurodevelopmental problems
Cohen Ronald A.; 2014 [72]		Male and female	Exposure to DBP	interfere with the thyroid function of pregnant women, since thyroxine (T4) and free T4 (FT4) were negatively associated with urinary levels of DBP	A reduction of thyroxine during prenatal life is associated with deficits in visual processing, visual–motor abilities, and motor skills, while a postnatal reduction is associated with deficits in language, weaker fine motor skills, lower attention, and lack of memory [64,65]
Daniel S. et al., 2020 [76]	411	Male	non-DEHP, sample phthalates collected during the third trimester of pregnancy	phthalates can disrupt hippocampal function and structure	anxious–shy behavior
		Female	DEHP metabolites, sample collected during the third trimester of pregnancy		decreased hyperactivity
Ponsonby A.L. et al., 2020 [77]	1074	Male and female	maternal Σ phthalate levels were 1.5 fold higher	Individual SNP interactions with phthalate levels were observed for cognition, ASD and ASD traits	offspring ASD and ASD traits

DBP: dibutyl phthalate; SNP: Single nucleotide polymorphism; ASD: autism spectrum disorder.

Phthalates also seem to negatively influence the intelligence of school-aged children, causing low intelligence quotient (IQ) scores at the age of 6–10 years old. There are also some indications that phthalate metabolism is associated with autism spectrum disorders (ASD) [77].

Furthermore, the association between phthalates exposure, dopamine system, and behavioral problems results rather interesting. It has been known for a long time how dopamine system dysfunction could have a key role in ADHD genesis [78,79]. Previous studies suggested that phthalates could disturb the release of dopamine in many different ways (e.g., interfering with dopamine receptor D2, tyrosine hydroxylase enzyme, and brain-derived neurotrophic factor (BDNF) expression [80,81]). Moreover, as mentioned above, phthalates increase the degrees of oxidative stress, of which dopaminergic neurons are particularly susceptible to [82]. 

The link between phthalates exposure and neurodevelopment effects needs more clinical longitudinal studies. The increasing amount of EDCs in our environment, due to plastic products and pollutants, leads to a continual and prolonged exposure for children. Pediatricians, but also schools and sport activities, should be informative and supportive for families and able to use precocious markers of neurodevelopmental disorders in their clinical practices.

## 4. Exposure to Phthalates Soon after Birth 

Newborns, particularly those recovered in Neonatal Intensive Care Units, are exposed to a wide range of phthalates: IV, feeding and suctioning tubing, plastic containers of IV fluids and medications, ventilation tubing and supplies, floors, walls, furniture, blood product containers and infusion systems, medical products such as hemodialysis, pheresis, extracorporeal membrane oxygenation (ECMO), respirator use, and IV fat emulsion. Neonates and infants are also exposed to phthalates through breastfeeding and maternal medications [45].

Several studies showed how breastmilk is an exposure route for phthalates to neonates. Calafat et al. [81] and Mortesen et al. [82] detected phthalates in breastmilk using the tandem mass spectrometry. They also analyzed a small sample of infant formula (based on cow’s milk) and consumer milk, and they found a large number of phthalates compounds in breastmilk rather than in the others, probably because cows are less exposed than humans. A complete opposite result has been found in [83]: a comparative analysis conducted on human milk and infant formula showed higher contamination of phthalates in artificial than in breastmilk. These findings highlight the need of wider investigations.

An Italian study confirmed that human milk may represent a source of phthalates exposure in infants; analyzing 62 samples of breastmilk from healthy women living in Southern Italy, mono-isobutyl phthalate and MEHP were detected in 100% of the samples, while mono-n-butyl phthalate (MBP) and monobenzyl phthalate (MBzP) were found in 64.5% and 43.5% of the tests, respectively. These data were confirmed also in women of other countries [84].

Women might be exposed to phthalates and may unknowingly pass them to neonates when breastfeeding. Personal care products (PCPs) have been proved to be a significant source of phthalates for the mother as well as for infancy during pregnancy. A recent study explored the association between urinary metabolite concentrations of BPA and phthalates in pregnant women, and the use of specific PCPs, food packaging, and medications [16]. The results highlight that higher levels of MEP concentration were found when make-up products and body lotions were used within the previous 24 h or if any PCPs were used in the previous 6 h. In addition, this study also analyzed the correlation among the usage of PCPs, different feeding types (breastfeed and formula), and urinary BPA and phthalates in infants: data showed that the phthalates’ levels in urine were higher in infants who used lotions or baby powder in the previous 24 h. 

Since PCPs undergo frequent formulation changes, further research in this field must be warranted [16].

Babies themselves do have a direct contact with phthalates in daily life. In fact, as they are inclined to hand-to-mouth activity and they consume greater food as a percentage of their body, they are exposed to a conspicuous quantity of plastic materials, such as in toys and in food packaging. As a consequence, infants and toddlers are the most vulnerable to phthalates exposure.

Everyday objects contain a small amount of phthalates, which is almost impossible to completely remove from babies’ lives. In fact, phthalates are found in cotton clothes that expose infants both to dermal-to-garment contact, which accounts for a minor issue, and to a more underestimated oral exposure [85]. Furthermore, phthalates are also present in paper diapers’ layers. Nevertheless, thanks to restrictive policies, the compounds’ presence is negligible [86].

Informative campaigns of sensitization should be considered to better inform the public opinion about this raising matter.

## 5. Conclusions

In recent years, several studies highlighted a relation between exposure to phthalates and endocrine and neurological development.

Complying with the recommendations of the International Scientific Societies, the majority of countries all around the world implemented a series of applicable and effective preventive measures ranging from specific policies to education of healthcare personnel and raising awareness throughout the general population, especially targeting pregnant women.

Since classic ortho-phthalates like DEHP have been proved to be toxic for reproduction and endocrine systems, the European Union (EU) and the United States of America have restricted the use of DEHP and other phthalates in toys and childcare [81]. Since 2015, the use of DEHP, DnBP, DiBP, and BBzP has been banned in the EU under the Registration Evaluation Authorization and Restriction of Chemicals (REACH) regulation with only few authorized exceptions [8,59], and over the last decade the US Consumer Products Safety Commission banned DEHP, DiBP, DBP, BBP, and DiNP from use in children’s toys and childcare articles in their Consumer Product Safety Improvement [10].

The decrease in the consumption volumes of DEHP in Europe and other part of the world mirrors the ongoing substitution process of this compound by toxicologically advantageous and less regulated alternatives. Among these compounds, there are di (2-ethylhexyl) terephthalate (DEHTP)—which is used as an alternative plasticizer in products like consumer goods, toys, food contact materials, and medical devices [87,88]—and di-iso-nonyl cyclohexane-1,2-dicarboxylate (DINCH), which has been used as an alternative plasticizer in medical devices, toys, and food packaging [8,86]. DEHTP and DINCH exposure has been shown to increase in the last years. However, epidemiological studies about DEHTP and DINCH exposure effects on human health are extremely limited [87].

Phthalates can impact different stages of human development. In particular, from the very beginning of life, they show a significant impact on steroidogenesis, modifying some endocrine endpoints, such as AGD.

Their influence does not stop there: analyzing the impact on puberty, data reported several results correlating phthalates prenatal exposure and early onset of puberty in girls [89].

Furthermore, other evidence showed the implication of phthalates in alterations of neurodevelopment. In particular, studies demonstrated that both the neuroendocrine system and the behavioral sphere are influenced by the phthalates concentrations. This branch of findings is in continuous development as more longitudinal studies are needed to have a more complete view on this issue.

The challenge for pediatricians is to work on the prevention side, making sure awareness programs are in place and working side by side with policymakers to have a systemic and multidisciplinary approach in tackling the spread of these compounds on the market. 

## Data Availability

Not applicable.

## Abbreviations

EDC	endocrine-disrupting chemicals
AGD	ano genital distance
DiDP	diisodecyl phthalate
DiNP	diisononyl phthalate
DHPH	di2-propylheptyl phthalate
DiUP	diisoundecyl phthalate
DTDP	diisotridecyl phthalate
PVC	polyvinyl chloride
LMW	low molecular weight
DBP	dibutyl phthalate
DiBP	diisobutyl phthalate
BBP	butyl benzyl phthalate
DEHP	2-Ethylhexyl phthalate
DMP	dimetyl phthalate
DEP	diethyl phthalate
DINCH	di-iso-nonyl cyclohexane -1,2-dicarboxylate
DEHTP	di (2-ethylhexyl) terephthalate
MBzP	monobenzyl phthalate
MBP	mono-n-butyl phthalate
MiBP	monoisobutyl phthalate
MEP	monoethyl phthalate
MEHP	mono(2-ethylhexyl) phthalate
